# Vitamins and Minerals for Energy, Fatigue and Cognition: A Narrative Review of the Biochemical and Clinical Evidence

**DOI:** 10.3390/nu12010228

**Published:** 2020-01-16

**Authors:** Anne-Laure Tardy, Etienne Pouteau, Daniel Marquez, Cansu Yilmaz, Andrew Scholey

**Affiliations:** 1 Sanofi Consumer Healthcare, Global Medical Nutritionals, 94250 Gentilly, France; Etienne.Pouteau@sanofi.com; 2 Sanofi Consumer Healthcare, 04000 México City, Mexico; Daniel.Marquez@sanofi.com; 3 Sanofi Consumer Healthcare, 34394 Beşiktaş Istanbul, Turkey; cansu.yilmaz@sanofi.com; 4 Centre for Human Psychopharmacology, Swinburne University, Victoria, VIC 3122, Australia; andrew@scholeylab.com

**Keywords:** B vitamins, vitamin C, iron, magnesium, zinc, energy production, mental and physical fatigue, anemia, cognition, mood.

## Abstract

Vitamins and minerals are essential to humans as they play essential roles in a variety of basic metabolic pathways that support fundamental cellular functions. In particular, their involvement in energy-yielding metabolism, DNA synthesis, oxygen transport, and neuronal functions makes them critical for brain and muscular function. These, in turn, translate into effects on cognitive and psychological processes, including mental and physical fatigue. This review is focused on B vitamins (B1, B2, B3, B5, B6, B8, B9 and B12), vitamin C, iron, magnesium and zinc, which have recognized roles in these outcomes. It summarizes the biochemical bases and actions of these micronutrients at both the molecular and cellular levels and connects them with cognitive and psychological symptoms, as well as manifestations of fatigue that may occur when status or supplies of these micronutrients are not adequate.

## 1. Introduction

The essential nature of vitamins and minerals for human health was demonstrated more than a hundred years ago. Recommendations for appropriate dietary intakes aim to ensure that most of the population receive amounts fulfilling their physiological needs [1]. The link between biochemical and physiological functions is established for some vitamins and minerals, as is their role in clinical outcomes. As an example, vitamin A is a component of the pigment rhodopsin located in the retina, that enables visual processes and prevents blindness. In many other instances, however, the nature of the involvement of micronutrients in molecular and cellular reactions that translate into physiological and functional effects is poorly understood. 

Claims regarding the effects of vitamins and minerals on fatigue, cognition or psychological functions are authorized in many countries [2]. However, the available scientific rationale for these is often based on theoretical biochemical grounds and clinical features seen during frank clinical deficiencies, rather than on robust, empirical, physiological data. Such clinical deficiencies are relatively uncommon, particularly in developed countries. Conversely, *subclinical* or inadequate intakes (sometimes referred to as ‘insufficiencies’) are frequent worldwide, albeit with variations according to age groups and country [3,4]. A varied and balanced diet, rich in nutrient-dense foods such as fruits, vegetables and dairy products, is able to provide the amounts of vitamins and minerals needed. There is ample evidence, however, that food choice or availability often preclude such a diet. This can lead to a significant proportion of the population not meeting their optimum dietary needs, in emerging and developed countries. For example, 68% of Mexican women have folate (vitamin B9) intakes below the estimated average requirement (EAR) [5], and thiamine (vitamin B1) dietary intakes are below EAR in 55% of Turkish adults of both genders [6]. Although the majority of Americans consume sufficient amounts of most nutrients to offset clinical symptoms, in many individuals intake falls below the EAR or Adequate Intake levels [7]. In such cases, vitamin and mineral supplementation may become a means to meet adequate intake. Indeed, this is one of the most frequent reasons for consumption given by supplement users. An enhanced feeling of well-being, a reduction in mental and physical fatigue and improvements in psychological and cognitive functions are also among the commonly reported motivations for taking supplements [8,9]. 

This narrative review aims to examine the scientific evidence that supports the role of key selected vitamins and minerals in health outcomes related to fatigue, as well as psychological and cognitive functions. Nine vitamins (vitamins B1, B2, B3, B5, B6, B9, B8, B12 and C) and three minerals (iron, magnesium and zinc) have been selected based on the health claims dealing with those health outcomes that have been authorized in Europe for these nutrients. Firstly, attention will be given to how the concept of energy is understood and how it can relate to physical and mental fatigue and performance. This section will briefly address both the biochemical/physiological perspective and the perceptual and psychological manifestations of energy. This is followed by a detailed, up-to-date review of the evidence for these micronutrients playing a role in physical and mental fatigue as well as in cognitive functions, focusing on biochemical pathways, with clinical information where available. Priority will be given to human data, especially those obtained in the healthy general population and in subjects with inadequate nutrient intake or status. Cognitive functions, as well as physical and mental fatigue, will be reviewed based on observed symptoms of deficiencies or subdeficiencies in human populations. Results from recent supplementation trials will also be considered. Most of the reported clinical data concern adult (or adolescent) populations. Some selected data on younger and older population will be included where relevant. 

## 2. Energy and Fatigue Are Subjective Perceptions Supported by an Objective Physiological Basis

### 2.1. Different Definitions Exist for Concepts Such as ‘Energy’ or ‘Fatigue’

From a nutrition science perspective, energy is provided by food, which is the only form of energy animals and humans can use to maintain the body’s structural and biochemical integrity. For the general public, energy is associated with the feelings of well-being, stamina and vitality that result in the ability to undertake their daily physical or intellectual activities and social relationships. Conversely, fatigue is often described as a perceived lack of energy or a feeling of low vitality [10]. 

Energy, vitality and fatigue are interrelated concepts, with the first two and the last sometimes seen as opposite ends of the same continuum (although they may be distinct psychological constructs [11]). Each can be defined as the sum of certain mental and physical components As examples the former may include degree of emotional and psychological well-being, level of perceived fatigue, mental resilience and perseverance, with the latter including feeling “strong and fit”, or “unable to do anything”) [12]. 

Similarly, fatigue descriptors often have both mental and physical aspects, with the former concerning both affective and cognitive functions. In an investigation of the experience of fatigue in 24 healthy men and women aged 24 to 72 years [13], lack of sleep or poor sleep was the major reported cause, while family and professional worries, stress and environmental factors (noise, heat, transportation) were also frequently cited. Physical aspects of fatigue included a lack of energy in two-thirds of subjects, with reduced stamina and a lack of strength reported in half of the cohort. Affective consequences of fatigue were reported as changes in mood and lack of motivation in 75% of subjects, low spirit and lack of vitality in 50%. In addition, more than half of subjects reported a diminished concentration and memory difficulties [13]. Similar conclusions were retrieved from a survey in a representative sample of the general Swedish population, who reported that both mental and physical fatigue were accompanied by a reduced activity level [14]. 

### 2.2. Fatigue, Lack of Energy and Lower Physical and Cognitive Performance

While the above-quoted surveys rely on subject report, their findings are consistent with more objective assessments of the effects of fatigue on mood and physical and cognitive performance. However, because current terminology does not reflect the wide range of fatigue states, and because validated experimental models are still missing, it is difficult to precisely describe and quantify how fatigue influences human performance [15]. Nevertheless, numerous studies have reported that fatigue elicited by physical training translates into worsened sport performance. For instance, cross-country skiers had decreased double poling performance after a 25 min trunk fatiguing exercise sequence, compared with a 25 min rest [16], and fatigued ballet dancers exhibited impaired movement control, which may increase the risk of injury [17]. Interestingly, impaired performance appeared to be associated with not only physical and muscular fatigue, but also mental fatigue. For example table tennis players hit the ball with lower speed and accuracy when they had previously completed a 90 min cognitive task [18].

Similarly, exhausting working conditions (long hours, shift work, stressful work environment) result in decreased cognitive performance. Fatigue from overnight hospital shifts resulted in worse diagnostic performance among radiologists, and the mean time to first fixate on a fracture increased by 34% among the fatigued physicians [19]. The link between fatigue and risk of road traffic accidents is well established, including in car drivers [20] and rail freight-regulating employees [21]. Similar deleterious effects are observed in in children, in whom fatigue is associated with poorer school performance [22,23,24]. 

### 2.3. What Are the Physiological Bases of Physical and Mental Fatigue

A common feature of fatigue is a “sense of energy depletion”, which can objectively be related to an insufficient amount of energy (calories). Mental and physical fatigue are experienced when these do not meet ongoing demands of the brain and muscles respectively. Under resting conditions, the fractional daily energy expenditure is the highest for heart and kidney (each approximately 440 kcal/kg), then for the brain (approximately 240 kcal/kg), then the liver (200 kcal/kg), while the resting skeletal muscle would need only approximately 13 kcal/kg. However, after considering the average adult weight of these organs, brain (1.33 kg) and muscle (26.3 kg) emerge as the most metabolically active structures, even at rest [25]. 

When considering physical activity, the contribution of muscle mass to energy demands further increases as a function of the intensity and duration of the exercise. Muscle adapts to such changes. Indeed, in spite of potentially large fluctuations in energy demand, available energy in the muscle remain globally constant, demonstrating a remarkably precise adjustment of the rate of the energy-generating processes to the demand. Limitation in energy supply is a classical hypothesis of muscle fatigue; it seems likely that limitations in the energy-generating processes indeed limit the rates of energy expenditure and hence performance [26]. Recent studies suggest that muscle fatigue may be the consequence of a metabolic challenge to a relatively small population of fast fatigue-sensitive muscle fibres, in which adenosine triphosphate (ATP) levels can be depleted to less than 30% of resting concentrations. As these fibres have a high contraction speed, they contribute greatly to power output, especially at faster movement rates, and depletion in available energy results in worsened performance and physical fatigue [27]. 

The adult human brain makes up only 2% of the body weight, but it consumes approximately 20% of the energy deriving from glucose, which provides the bioenergetic basis for neurotransmission. The proportional energy need of the adult human brain is more than twice that of adult non-primate vertebrates, which allocate only 2% to 8% of their basal metabolism to their brain; this may be related to the higher development and larger neuronal number of the human brain [28]. Indeed, most of the energy consumed by the brain is dedicated to neuronal functioning. Synapse transmission (e.g., the process enabling communication between neurons, which involves conversion of an electric action potential into a chemical message [neurotransmitter]) is a highly energy-demanding process. Large amounts of ATP are dedicated to maintaining the resting energy potential and to manage neurotransmitter synthesis and processing within synapses [29]. Unlike the skeletal muscles, which contain significant amounts of stored energy in the form of glycogen, the brain has only minimal energy reserves and it is thus dependent on the regular supply of energy substrates from the circulation through the blood–brain barrier. Under normal physiological and nutritional conditions, the major energy fuel for the brain is glucose. However, when glucose availability is reduced, the brain can use alternative energy substrates such as lactate, medium-chain triglycerides and ketone bodies [30]. 

Another important aspect of the brain is that, unlike muscle, it is always highly metabolically active. Intrinsic electrochemical activity occurs continually, including during sleep, and mental work thus only adds a small increment (less than 5%) to the basal neural activity. In other words, the brain continuously consumes a considerable amount of energy, even in the absence of any particular task [31]. While this is true for the whole brain, local neuronal activity can change significantly, during sensory or motor stimulation and also during the period that separates sleep and waking situations. In addition, memory formation and synaptic plasticity are accompanied by changes in neuronal communication/firing. A phenomenon known as ‘neurometabolic coupling’, adjusts the local energy supply and cerebral blood flow to variations in neuronal activity and enables appropriate metabolic substrate delivery to maintain functioning of neurons [32]. 

### 2.4. Fatigue May Also Result from A Dysfunction in The Supply of Oxygen to Muscles and Brain

Brain and muscle tissue are both also highly dependent on oxygen. The brain consumes approximately 3.5 mL O_2_ per minute and per 100 g of tissue, which corresponds to 20% of the oxygen needs of the whole body. Compromised oxygen delivery can harm the brain, with chronic hypoxia resulting in impaired intellectual function [33]. Resting muscle consumes ‘only’ 1 ml O_2_ per minute and per 100 g of tissue, but oxygen consumption can increase up to 50-fold in contracting muscle, when exercising [34], and this enables enhanced performance [35]. During anemia, i.e., when the levels of hemoglobin (the oxygen carrier) are decreased, oxygen delivery is impaired, with consequences for not only cognitive [36] and physical [35] performance, but also perceived fatigue and tiredness [37]. It is notable that sustained, intense mental processing results in a measurable decrease in circulating levels of glucose [38,39,40] and oxygen [41]. Fatigue is considered a major component of anemia symptomatology, whatever the underlying reason for decreased hemoglobin level [42]. 

## 3. Vitamins and Minerals Have Critical Roles in Cellular Energy Production

### 3.1. Overview of Cellular Energy Production

In humans, dietary macronutrients provide the fuel required to maintain the biochemical and structural integrity of the body, to perform physical activity and to enable new tissue deposition [43]. Ingested food is digested by enzymes that break down carbohydrates into monomeric sugars (monosaccharides), lipids into fatty acids and proteins into amino acids. Sugars, fatty acids and amino acids enter the cell, where a gradual oxidation occurs, first in the cytosol, then in the mitochondria. The energy-generation process can be broken down into the three steps described below, that ultimately produce chemical energy as ATP that can be easily used elsewhere in the cell. 

**Figure 1:**
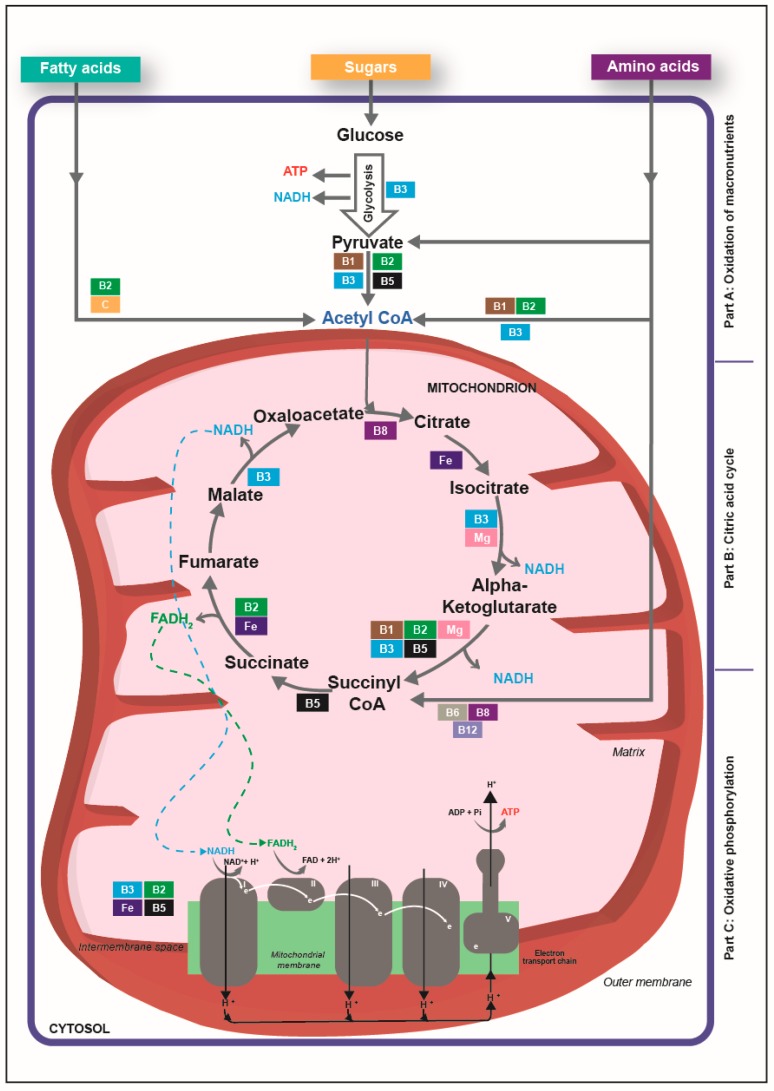
Overview of the involvement of vitamins and minerals in the major pathways of cellular energy production. This figure displays a simplified scheme of energy metabolism. Briefly, macronutrients are oxidized (part A) into acetyl-CoA through several pathways including glycolysis, which produces pyruvate from glucose, and vitamins B1, B2, B3, B5 and C play important roles Then acetyl-CoA enters the citric acid cycle (part B), which generates energy as NADH and FADH_2_ through a series of eight oxidations that involve vitamins B1, B2, B3, B5, B6, B8 and B12 as well as iron and magnesium. Finally, the electrons of NADH and FADH_2 _ are transferred to the electron transport chain (part C), where they provide energy used to generate ATP molecules; this step needs the input of vitamins B2, B3, B5 and of iron.

Oxidation of macronutrients into acetyl co-enzyme A (acetyl-CoA, Figure 1, part A)

Acetyl-CoA is an activated carrier molecule that is derived from pyruvate that is itself issued from glucose during glycolysis, from fatty acids through beta-oxidation, and from certain amino acids (although they are preferably spared for protein synthesis) [44].

Citric acid cycle (Figure 1, part B)

Within the mitochondria, acetyl-CoA is transferred to oxaloacetate, a 4-carbon molecule, to form the 6-carbon citric acid. Citric acid is then gradually oxidized across eight reactions that generate energy, stored in three molecules of nicotinamide adenine dinucleotide (NADH) and one molecule of reduced flavin adenine dinucleotide (FADH_2_), which both are activated electron carriers [44]. 

Oxidative phosphorylation (Figure 1, part C)

The inner mitochondrial membranes contain an electron transport chain consisting of five protein complexes, among which three (complexes I, III, and IV) pump protons (H + ) to generate a H+ gradient for ATP production at complex V [44]. NADH and FADH_2_ transfer their electrons to the electron transport chain. As they proceed through this chain, electrons lose their energy, which is used to generate ATP by the phosphorylation of adenosine diphosphate (oxidative phosphorylation). The resulting low-energy electrons are combined with oxygen molecules and with protons (H +) from the surrounding solution to produce water [44].

Roughly a billion molecules of ATP are in solution in a typical cell at a given time and, in most cells, this ATP is used and replaced every 1 to 2 minutes [44]. This complex and highly efficient system exploits the energy-containing macronutrients as well as the vitamins and minerals that make possible the extraction of energy from these macronutrients.

### 3.2. The Interplay of B Vitamins in Cellular Energy Production

All the B vitamins except folate are involved in at least one and often in several steps of the energy-production system within the cell (Figure 1). Adequate supply of each B vitamin is required for appropriate functioning of the energy-production system and a shortfall in any one of them will be rate limiting for energy production, with potentially severe metabolic and health consequences. 

Vitamin B1 occurs in the body as free thiamine and as various phosphorylated forms, including thiamine pyrophosphate (TPP). TPP is involved in dehydrogenase reactions, which result in the decarboxylation of pyruvate and of branched-chain amino acids to form acetyl-CoA (Figure 1, part A). Within the citric acid cycle, TPP supports the decarboxylation of alpha-ketoglutarate into succinyl-CoA [45,46] (Figure 1, part B). 

Riboflavin (vitamin B2) is an integral part of the coenzymes flavin adenine dinucleotide (FAD) and flavin mononucleotide (FMN), which are mandatory in the function of the ‘flavoprotein enzymes’. FAD and FMN act as proton carriers in redox reactions critical for the metabolism of carbohydrates, fats, and proteins [47]. FAD is involved in the production of acetyl-CoA from fatty acids via beta-oxidation, from glucose via the oxidative decarboxylation of pyruvate and from catabolism of branched amino acids (Figure 1, part A). FAD is required in the citric acid cycle steps that produce succinyl-CoA from alpha-ketoglutarate and fumarate from succinate (Figure 1, part B), and FADH_2_ acts as an electron donor in the electron transport chain (Figure 1, part C). 

Niacin (vitamin B3) refers to nicotinamide and derivatives that exhibit the biological activity of nicotinamide, which is a precursor for NAD and nicotinamide adenine dinucleotide phosphate. During glycolysis, electrons are removed by NAD+ (producing NADH) from some of the carbons derived from the glucose molecule (Figure 1, part A). NAD is also required in the citric acid cycle steps that produce isocitrate from succinyl-CoA and then malate to oxaloacetate (Figure 1, part B). Finally, mitochondrial oxidative phosphorylation starts in complex I of the electron transport chain, where NADH is oxidized and donates its electron to initiate transport across the chain (Figure 1, part C) [45].

Pantothenic acid (vitamin B5) is an essential precursor in the biosynthesis of Coenzyme A, which is at the crossroads of cellular metabolism as it reacts with acyl groups (Figure 1, part A). It gives rise to thioester derivatives, including acetyl-CoA and succinyl-CoA, which feature in the citric acid cycle (Figure 1, part B). 

Pyridoxine (vitamin B6) refers to a group that includes pyridoxine, pyridoxal and pyridoxamine, and their respective phosphorylated forms. The metabolically active forms act as cofactors of enzymes involved in amino acid metabolism, one-carbon reactions, glycogenolysis and gluconeogenesis, heme synthesis, and niacin formation from tryptophan, as well as in lipid metabolism and hormone action [48]. Pyridoxal phosphate (PLP) is a cofactor for glycogen phosphorylase, which releases glucose-1-phosphate from glycogen and provides additional glucose when needed, such as in exercising muscle (not shown on figure) [48].

Biotin (vitamin B8) serves as a cofactor for several carboxylases that catalyze the incorporation of bicarbonate as a carboxyl group into a substrate. These enzymes play critical roles in the synthesis of fatty acids, the catabolism of branched-chain amino acids and gluconeogenesis and are thus critical for energy production and storage within the cell [49,50]. The major biotin-dependent enzymes involved in energy production are propionyl-CoA carboxylase, which catalyzes the incorporation of bicarbonate into propionyl-CoA to form methylmalonyl-CoA, which enters the citric acid cycle through conversion to succinyl-CoA, and pyruvate carboxylase, which catalyzes the carboxylation of pyruvate to form oxaloacetate, an intermediate in the citric acid cycle (Figure 1, part B). Other biotin-requiring enzymes regulate the availability of fatty acids for mitochondrial oxidation or amino acids for use in the citric acid cycle. 

Cobalamin (vitamin B12) is a generic term for a group of compounds known as corrinoids that exhibit qualitatively the biological activity of cobalamin. One of these active corrinoids is 5’-deoxyadenosylcobalamin, which is the cofactor of the methylmalonyl-CoA mutase enzyme. This enzyme catalyzes the conversion of methylmalonyl-CoA into succinyl-CoA (Figure 1, part B). This conversion occurs during the oxidation of odd-chain fatty acids and the catabolism of ketogenic amino acids [51]. 

### 3.3. Vitamin C, Iron and Magnesium Are Also involved in Energy-Yielding Metabolism

Vitamin C is needed for two dioxygenase enzymes involved in the biosynthesis of carnitine, an essential cofactor in the transport of long-chain fatty acids into the mitochondria. It thus plays an important role in the production of energy via beta-oxidation (Figure 1, part A) so impaired carnitine metabolism, including through insufficient vitamin C supply, can be responsible for weakness or muscle aching [52,53].

Iron is included within the porphyrin ring structure of heme enzymes, such as for the family of cytochromes required for cellular energy production. Cytochromes serve as electron carriers during the synthesis of ATP in the electron transport chain: the reduction of ferrous iron to ferric iron is coupled with the acceptance of electrons. Among the 40 different proteins that constitute the respiratory chain, there are six different heme iron proteins and six others with iron-sulphur, located in complexes I, II and III (Figure 1, part C) [54]. Among those, succinate dehydrogenase is as a key enzyme in the citric acid cycle (Figure 1, part B).

Magnesium has a predominant role in the production and utilization of ATP. Indeed, each molecule of ATP binds to a magnesium ion (Mg^2 +^) to compose its biologically functional form: in the cell, most of the ATP is present as Mg-ATP complexes. Such complexes are, for example, cofactors for several kinases active during glycolysis (Figure 1, part A). Magnesium acts also as a regulator of the activity of several enzymes of the citric acid cycle, including the isocitrate dehydrogenase and the oxoglutarate dehydrogenase complex (Figure 1, part B) [55]. Furthermore, ATP binds to magnesium ions to compose biologically functional forms and, in the mitochondria, ATP–Mg complexes help export mitochondrial ATP into cytosol and thus deliver energy within the cell [56,57,58].

## 4. Vitamins and Minerals Are Important for Regulating Oxygen in the Body

### 4.1. Oxygen Transport Requires Iron and Vitamins B6, B9 and B12 

Approximately two-thirds of the iron in the body is found in hemoglobin, a heme-containing protein concentrated in red blood cells. Heme iron is present in its ferrous state (Fe^2+^), enabling a reversible binding of oxygen. One hemoglobin molecule can thus transport four oxygen molecules, and as a result blood carries 50–70 times more oxygen than would plasma alone. The vital role of hemoglobin is derived from the unique ability provided by iron to acquire oxygen rapidly during the transient period in contact with the lungs, and to release oxygen as needed during its circulation through the tissues [54]. Myoglobin, another heme protein, allows the transport and short-term storage of oxygen in muscle cells, helping to match the supply of oxygen to the high demand of working muscles. Iron deficiency anemia reduces blood transport and supply of oxygen to muscle, impairing endurance capacity and energetic efficiency [54]. 

**Figure 2:**
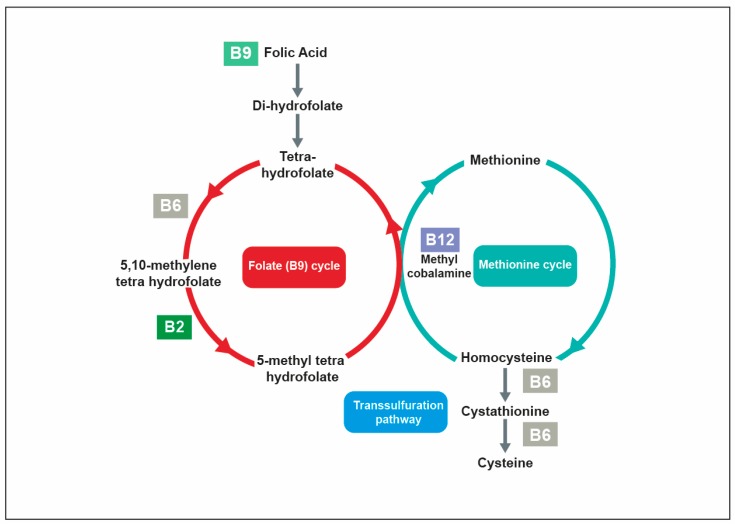
Metabolic and functional interactions of vitamins B9, B12 and B6.

Anemia may have other nutrition-related origins than iron deficiencies and there is evidence that inadequate supplies of certain B vitamins, primarily vitamins B6, B9 and B12, can lead to anemias. Metabolic and functional pathways of vitamins B9 and B12 and, to a lesser extent, vitamin B6 are indeed closely interrelated (Figure 2). The folate cycle, which is critical to the generation of the active forms of vitamin B9, is dependent upon vitamin B12. In its methylcobalamin form, B12 is itself mandatory for the activity of the enzyme methionine synthase, which helps in coupling the demethylation of tetra-hydrofolate and the synthesis of methionine from homocysteine by the transfer of a methyl group [51]. Vitamin B6 is also involved in homocysteine catabolism, through the transsulphuration pathway, which converts homocysteine into cysteine via two vitamin B6 (PLP)-dependent enzymes. A deficiency in vitamin B9, B12 or B6 can thus lead to the accumulation of homocysteine [59]. 

Blood-borne oxygen transport in the blood depends on vitamin B6, as PLP is the cofactor of alpha-amino levulinate synthase, an enzyme needed for the synthesis of the porphyrin ring of hemoglobin. Chronic deficiency in vitamin B6 may trigger microcytic anemia, characterised by a low concentration of hemoglobin in erythrocytes [48]. 

Folate (vitamin B9) functions as a cofactor or co-substrate in many one-carbon transfer reactions that are important for amino acid metabolism and for the synthesis of nucleic acids [60]. When DNA synthesis is impaired, the production of red blood cells, a process requiring intense cell replication, is disrupted and deficiency in folate can lead to megaloblastic anemia, characterised by a low red blood cell count and an accumulation of red blood cell precursors in the bone marrow, in the form of large, immature, nucleated megaloblasts, all of which lowers the oxygen transport capacities of blood [61]. 

Because of the interdependence of folate and cobalamin (vitamin B12) metabolism (Figure 2), megaloblastic anemia is also a frequent clinical expression of cobalamin deficiency (present in 70–80% of cases of deficiency). Indeed, DNA synthesis is impaired by low vitamin B12, which hampers the activation of folate and, as a result, decreases normal red blood cell production, impairing oxygen delivery [62]. 

### 4.2. Riboflavin, Vitamin C, Iron, Magnesium and Zinc and Oxidative Stress 

Brain and muscle cells need oxygen, for aerobic metabolism, but oxygen is simultaneously involved in the genesis of degenerative states because of the high reactivity of oxygen free radicals [63]. This is especially the case in tissues such as the muscle or brain, which consume large amounts of oxygen. The organism has developed a powerful antioxidant defence system in which certain vitamins and minerals play an important role. 

Riboflavin (vitamin B2) has a specific and major protective role against lipid peroxides, provided by its involvement in the glutathione (GSH) redox cycle. GSH is a potent antioxidant, thanks to its thiol group, which acts as an electron donor. Once oxidized, GSH can be regenerated by the FAD-containing enzyme GSH-reductase, back to a form that is again able to act against free radicals. Furthermore, riboflavin deficiency is known to decrease the activity of glucose 6-phosphate dehydrogenase, an enzyme that is also involved in the GSH cycle [64]. 

Vitamin C refers to both ascorbic acid and L-dehydroascorbic acid. Ascorbic acid is readily oxidized to L-dehydroascorbic acid, which, in turn, can be reduced back to ascorbic acid. This proceeds through transfer of electrons, and vitamin C is thus an electron-provider for other molecules: it functions as a supplier of ‘reducing equivalents’, being itself oxidized during this transfer. This role of reducer (therefore, by definition, an "antioxidant") can be exercised in a non-specific or specific way and can explain the multiple functions and effects of vitamin C [52,53]. Because of this ability to donate electrons, ascorbic acid scavenges reactive oxygen species as well as singlet oxygen. High tissue levels of ascorbate provide substantial antioxidant protection where free radicals are encountered [52,53]. Because of its protective role versus oxidation, vitamin C deficiency in the brain may cause oxidative stress and neurodegeneration, related to the high oxygen demand of brain structures [65]. Vitamin E prevents the propagation of a chain reaction of lipid oxidation that could especially affect polyunsaturated fatty acids in the membrane, including in neuronal structures [66]. When a molecule of vitamin E neutralizes a free radical, it is oxidized and its antioxidant capacity is lost. Other antioxidants, such as vitamin C, are capable of regenerating the antioxidant capacity of vitamin E within a chain of oxidative-reductive reactions [67].

Although magnesium does not, strictly speaking, belong to the antioxidant defence system [68], there is ample evidence that a lack of magnesium leads to oxidative stress [69,70]. Among the suspected mechanisms, inflammation associated with low magnesium levels is known to increase the production of free radicals in phagocytes and neutrophils and lead to endothelial dysfunction [68]. Preclinical studies have also shown that magnesium deficiency leads to increased nitric oxide production, which, when in excess, can trigger the formation of reactive oxygen species, such as hydrogen peroxide [71]. 

A body of evidence supports the anti-oxidative properties of zinc, which can act by inhibition of oxidation of macromolecules such as nucleic acids and proteins. Zinc interacts with several enzymes that are critical in the antioxidant defence system: it induces the expression of metallothionein and increases the activity of catalase, both of which can scavenge reactive oxygen species. Zinc is a cofactor of superoxide dismutase, which neutralizes superoxide anions to produce hydrogen peroxide, which is further metabolized by catalase [72,73]. The anti-inflammatory role of zinc eventually results in the down-regulation of the production of reactive oxygen species: for example, zinc is able to modulate the activity of the nuclear factor-κB signalling pathway and is thus able to indirectly affect the expression of numerous genes involved in immune and inflammatory responses [74]. 

Despite iron being mandatory for oxygen transport and other functions, excess iron may be toxic to cells because it can promote the generation of free radicals via the Fenton’s reaction, in which iron reacts with hydrogen peroxide and produces hydroxyl radicals, with potentially highly deleterious consequences [54], especially in the brain, where iron-induced oxidative stress may cause neurodegenerative processes [75,76]. This pro-oxidative action of iron is however limited, in normal physiological conditions, by the very tight regulation of iron metabolism via homeostasis, which prevents iron overload. Moreover, free iron, the most reactive form, is not physiologically present in the body, as iron is bound to transferrin and ferritin in the plasma and to several chaperones and iron-binding proteins in cells, reducing the chances of free or nonligated iron becoming available to participate in the Fenton reaction [77,78].

## 5. Vitamins and Minerals Are Critical for the Structure and Function of Brain Cells 

Besides the fundamental roles vitamins and minerals in contributing meeting play by contributing to the high demand of energy from the brain, these micronutrients are also important to establish and maintain brain structures and to enable intercellular connections (i.e., his is true for healthy subjects, of all ages [79], but is especially critical during infancy and young childhood, when brain development occurs [80], and during ageing, when significant structural and functional changes in the brain occur [81]).

### 5.1. Vitamins and Minerals Are Involved in Neuronal Structures 

Thiamine (vitamin B1) is involved in the formation of synapses, the growth of axons and myelin genesis, leading to the establishment of a functional neuroglia. It is also able to stabilize the membrane of newly generated neuronal cells during embryogenesis and may control apoptosis (programmed cell death); this may proceed through suspected thiamine-binding sites, present on biological membranes [82]. 

Pantothenic acid (Vitamin B5) is an essential precursor in the synthesis of acetyl-CoA. Many soluble proteins are acetylated (i.e., an acetyl group is substituted for an active hydrogen atom) by acetyl-CoA at their N-termination. N-Acetylation is one of the most common covalent modifications of proteins, crucial for their regulation and function, and approximately 85% of all human proteins are acetylated [83]. These post-translation modifications are in particular present in nervous system structures: protein acetylation also appears important for neuronal development [84].

Folate (vitamin B9) is involved in cerebral methylation processes and is important in maintaining neuronal and glial membrane lipids, which could have effects on more general brain functions as reflected in changes in mood, irritability and sleep [85,86].

Several components of the nervous system are modulated by the concentrations in ascorbate (vitamin C), including neurotransmitter receptors and brain cellular structures (such as glutamatergic and dopaminergic neurons) and the synthesis of glial cells and myelin [65,87].

Iron is known to be critical for neuronal differentiation and proliferation. Iron deficiency affects neural processes such as myelination, dendritic arborization and neural plasticity [88]. 

Finally, zinc is considered essential for the formation and migration of neurons and for the formation of neuronal synapses [89]. 

### 5.2. The Synthesis of Neurotransmitters Is Dependent on Vitamins B1, B5, B6, B9 and C

Thiamine (vitamin B1) is required for the synthesis of fatty acids, steroids, nucleic acids and aromatic amino acids, which are precursors to a range of neurotransmitters, including acetylcholine, glutamate and gamma-aminobutyric acid [90].

Pantothenic acid (vitamin B5) is required for the synthesis of the neurotransmitter acetylcholine, as shown by experimental studies in rats in which pantothenic acid was depleted using chronic alcohol exposure that displayed a decreased synthesis of acetylcholine in the brain [91]. 

In the brain, the aromatic L-amino acid decarboxylase, an enzyme dependent on PLP (vitamin B6), catalyzes the synthesis of two major neurotransmitters: serotonin from tryptophan and dopamine from phenylalanine. The synthesis of other neurotransmitters, including glutamate or gamma-aminobutyric acid, is also catalyzed by enzymes that require vitamin B6 as cofactors [92]. 

Vitamin B9 enables cerebral methylation processes and this affects the metabolism of the neurotransmitters serotonin and dopamine, which are important in mood regulation. More precisely, folate has been linked to the maintenance of adequate cerebral levels of tetrahydropterin, a key cofactor in the hydroxylation reactions that lead to the synthesis of serotonin and catecholamines. [85,93]. 

The synthesis of neurotransmitters is also dependent on methionine, as a precursor to S-adenosyl-L-methionine, a universal methyl donor active in numerous metabolic pathways related to the synthesis of hormones, neurotransmitters, nucleic acids, and proteins, particularly in the brain. The metabolic interplay between vitamins B6, B9 and B12 (Figure 2) is thus important to enable an appropriate provision of methionine [94].

Vitamin C is involved in the synthesis and modulation of some hormonal components of the nervous system. It is a cofactor of the enzymes that catalyze the formation of catecholamines: (noradrenaline and adrenaline), and of enzymes that are active in the biosynthesis of neuropeptides [65]

### 5.3. Vitamins B3 and B5, Iron, Magnesium and Zinc Are Important for Neurotransmission 

Through NAD, niacin (vitamin B3) is involved in the control of intracellular calcium release. Calcium signalling is strongly integrated with nucleotide metabolism and the energy status of the cell, which are both dependent on NAD. Knowing the role of calcium in generating action potential in neurons, this is of high relevance regarding the role of niacin in cerebral functions [95]. 

Pantothenic acid-dependent palmitoylation of certain neuronal proteins is needed for release of neurotransmitters in the synapse, and is thus mandatory for transduction of information in the brain [83]. 

Iron is thought to be important for synaptic function as iron deficiency has been shown in a large set of preclinical studies to induce alterations in the electrophysiological properties of neural circuitry and neurotransmitter systems [88]. Furthermore, recent findings suggest that a limited and controlled amount of reactive oxygen species in hippocampal neurons, including those generated by iron through the Fenton reaction, may stimulate calcium release, and thus allow calcium signals to activate the signalling cascades that lead to the transcription of genes known to participate in synaptic plasticity [96].

Magnesium is involved in the active transport across cell membranes of potassium and calcium. In the nervous system, magnesium is thus important for neuromuscular coordination and optimal nerve transmission. It is also useful to protect against excessive excitation leading to cell death: it interacts with the aspartate receptor, by blocking the calcium channel in this receptor, and must be removed for glutamatergic excitatory signalling to occur. Low magnesium levels may theoretically increase glutamatergic neurotransmission, which can lead to oxidative stress and neuronal cell death [97].

High concentrations of zinc are present in the synaptic vesicles of specific neurons, called ‘zinc-containing’ neurons, where it has a major role in controlling synaptic excitability by modulating neurotransmission mediated by both glutamate and gamma-aminobutyric acid. Zinc is also potently neurotoxic and could increase neuronal death during transient global ischemia and brain trauma, but neurons have numerous homeostatic systems to maintain zinc concentrations at levels that are nontoxic [89]. 

## 6. Clinical Evidence of the Role of Vitamins and Minerals on Physical Fatigue

When vitamins and minerals are supplied in adequate amounts to humans, their biochemical properties translate into normal physiological functions. When the supply is below requirements, frank deficiencies may occur, that manifest themselves with clinical symptoms. There is growing evidence that intermediate situations, often referred to as ‘suboptimal’ or ‘inadequate’ nutrient status, ‘marginal deficiency’ or ‘insufficiency’ may be associated with subclinical functional deficits and/or with enhanced risks of pathologies. Both clinical and subclinical deficiencies can be addressed by nutrient supplementation [98].

### 6.1. Impact of Inadequate Status of Vitamins and Minerals on Physical Fatigue 

Frank deficiencies in most vitamins and minerals have been associated with lethargy or physical fatigue, which can also be observed in marginal deficiencies, but these symptoms are often missed because they are nonspecific. 

#### 6.1.1. Inadequate Status of Individual B Vitamins and Physical Fatigue

Beriberi, the disease resulting from severe thiamine (vitamin B1) deficiency affects several organs, including the muscular and peripheral nervous systems [99]. General symptoms include fatigue, ataxia due to muscle weakness in the legs and arms, muscle pain and tenderness, and dyspnoea on exertion. 

Riboflavin (vitamin B2) deficiency is most often accompanied by other nutrient deficiencies and it is thus difficult to clearly identify specific symptoms. Anemia, a condition related to fatigue, is associated with low riboflavin status. For example, in 1253 adult Chinese individuals followed for 5 years, more than 97% had inadequate riboflavin intake at baseline and this was associated with an increased risk of anemia at follow-up [100]. High rates of marginal and deficient riboflavin status have been found in healthy women from both Canada and Malaysia and were indicative of a higher risk of anemia [101], which enhances the risk of associated fatigue symptoms.

Inadequate niacin (vitamin B3) intakes or status lead to nonspecific clinical symptoms and include weakness, loss of appetite, fatigue and apathy [102].

Vitamin B5 deficiency has been experimentally induced using a pantothenic acid kinase inhibitor and a pantothenic acid deficient diet. After approximately 6 weeks, subjects complained of headache, fatigue, insomnia, and they reported excessive fatigue after their daily walk; symptoms decreased then disappeared after 4 weeks of supplementation with 4 g pantothenic acid daily [103]. One case of lethargy, associated with anorexia, weight loss, and hypochromic anemia, has been described, in a middle-aged woman. She experienced these symptoms twice, with an interval of several years, and they improved dramatically on each occasion following administration of pantothenic acid [104].

One of the most typical features of pyridoxine (vitamin B6) deficiency is microcytic anemia, due to defective hemoglobin biosynthesis and characterised by symptoms of weakness, tiredness or fatigue [105]. The importance of maintaining an adequate vitamin B6 status has been highlighted by the observation that iron supplementation could be ineffective in treating iron deficiency anemia in pregnant women who had vitamin B6 deficiency [106]. Similar conclusions had been made earlier from a study in iron-deficient German children who had an accelerated hemoglobin synthesis and thus recovered better from anemia after an 8 day period when they had been treated with a combination of iron and vitamin B6, compared with iron alone [107]. 

Deficiencies in folate (vitamin B9) can result in megaloblastic anemia, which produces symptoms of weakness and fatigue, headache, palpitations, and shortness of breath. Weakness and fatigue typically appear at an advanced stage, but may be seen at milder degrees of anemia in some patients, especially the elderly [108].

Deficiencies in cobalamin (vitamin B12) can result in diminished energy and exercise tolerance, together with fatigue and shortness of breath. These hematologic symptoms regress and disappear with supplementation with vitamin B12, for which doses and routes will depend on the cause and severity of deficiency [109]. 

#### 6.1.2. Inadequate Status in Vitamin C and Physical Fatigue

Before the characteristic features of scurvy (the disease caused by severe vitamin C deficiency) are present, symptoms of moderate deficiency include fatigue, irritability, and muscle pain. 

The association between plasma vitamin C level and physical functional health was evaluated in the general UK population [110]. More than 15,000 healthy men and women, aged 40 to 79, had their blood vitamin C measured and completed the well-validated 36 Item Short-Form Health Survey (SF-36) questionnaire. The SF-36 measures self-reported physical and mental health across a number of domains, including those related to physical health and vitality. The population was sorted in quartiles, according to their blood vitamin C levels, from the lowest (below 41 µmol/L) to the highest (above 66 µmol/L) and risk of poorer outcomes was estimated using regression models that adjusted results according to different potential confounding factors (sociodemographic factors, lifestyle factors, and comorbidities). Whatever the regression model, those in the lowest quartile of vitamin C, compared with the highest, had significantly increased odds of having a poor physical functional health SF-36 score, a poor bodily pain score, a poor self-reported general health and a poor vitality score. When vitamin C supplementation was considered as a stratification factor, the results were no longer statistically significant for a few physical functional health domains in those who received vitamin C supplementation, while the results became more consistent for participants with no vitamin C supplementation [110]. This study is consistent with the previous finding that high intakes of fruits and vegetables (the major source of vitamin C in the diet) were associated with better self-reported physical functional health [111].

#### 6.1.3. Consequences of Inadequate Status in Iron and Magnesium on Physical Fatigue

The pallor of anemia was associated with weakness and tiredness long before its cause was known. Common symptoms of iron deficiency in adults include fatigue and low resistance to exertion. These symptoms can be related to low delivery of oxygen to body tissues and decreased activity of iron-containing enzymes. Numerous studies report fatigue and weakness when anemia due to iron deficiency is present, i.e., when hemoglobin levels are below 120 g/L in women or 130 g/L in men, and also during iron deficiency without anemia, i.e., with normal or borderline hemoglobin levels [112]. Besides increasing perceived fatigue, iron deficiency is also known to decrease physical performance, work efficiency and working capacity [113]. When addressing aerobic capacity (measured by VO_2_max, i.e., the maximum amount of energy consumed by a subject during an intense physical exercise), evidence from clinical studies suggests that both severe and moderate iron deficiency anemia impair aerobic capacity, and this impairment is in proportion to the severity of anemia. Impairments can disappear by increasing hemoglobin concentration via iron supplementation [113]. 

Most of the early symptoms of magnesium deficiency include neurological or neuromuscular manifestations. Neuromuscular hyperexcitability, including muscle cramps, is a common feature of magnesium deficiency, but latent tetany, generalized seizures, vertigo and muscular weakness may also be present [56]. These symptoms are related to the role of magnesium in nerve transmission and muscle contraction. Other symptoms of magnesium deficiency may include fatigue, lethargy, staggering, and loss of appetite [114].

Significantly more studies have addressed the role of magnesium on physical performance. Magnesium deficiency has been shown to impair physical performance, while several cross-sectional surveys have reported a positive association between magnesium intake or serum magnesium concentrations and outcomes related to muscle strength, muscle performance or muscle power [115]. For instance, one small cross-sectional study performed in 26 male athletes indicated a positive and significant association between magnesium intake and maximal isometric trunk flexion, rotation, hand grip, jumping performance, and isokinetic strength indicators [116]. Exercise has an impact on magnesium distribution and utilization (with magnesium being transported to locations where energy production is taking place, in response to exercise), and magnesium is necessary for muscle contraction and cardiorespiratory functions [115]. Furthermore, since magnesium needs are likely to be increased in conditions where the metabolism is accelerated, physically active subjects may have higher magnesium requirements. Suboptimal or deficient magnesium status in subjects involved in strength training programmes may be associated with less efficient energy metabolism and impaired endurance capacity. On the contrary, higher magnesium intakes have been reported to be associated with lower oxygen needs and better cardiorespiratory indices in aerobic exercise [115,117]. 

### 6.2. Effects of Supplementation with Vitamins and Minerals on Physical Fatigue

Few studies have addressed the effect of supplementation with vitamins and minerals on physical fatigue and data are lacking, especially regarding niacin, pyridoxine or cobalamin. Existing supplementation studies most often use large doses, well above daily nutritional requirements: this helps provide the proof of concept and demonstrates the role of the vitamin or mineral in alleviating physical fatigue but it does not document the appropriate amounts to be given or the nutrient status that should be reached. 

#### 6.2.1. Supplementation with Individual B Vitamins and Physical Fatigue 

In 16 young athletes, high-dose thiamine (vitamin B1) supplementation (100 mg/day) for 3 days markedly increased blood thiamine level and significantly decreased the number of complaints after a cycling exercise, in a subjective fatigue assessment [118]. 

Improving riboflavin (vitamin B2) nutritional status has been found to increase circulating hemoglobin levels and improve anemia. In 119 young British women, aged 19–25 years, who were mildly deficient in riboflavin, daily supplementation with 2 or 4 mg riboflavin for 8 weeks resulted in an increase in blood hemoglobin, which was correlated to the improvement in riboflavin status. The latter increased from 0.56 nmol/L to 13.86 and 29.49 nmol/L respectively, after 2 and 4 mg vitamin B2 daily [119]. A recent original study has addressed symptoms that can be connected to physical fatigue, due to muscle pain and soreness after a physical exercise. Thirty-two adults (80% men) who participated in an ultramarathon (161 km race) received riboflavin (100 mg) or a placebo capsule shortly before the race start and again when reaching 90 km. Muscle pain and soreness ratings during and immediately after the race were found to be significantly lower for the riboflavin-treated group. When performance on 400 min runs was measured, 3 and 5 days after the ultramarathon, times were significantly faster for the riboflavin-treated group. Although preliminary, this work suggests that riboflavin supplementation before and during prolonged running may have a beneficial effect on muscle fatigue [120].

A few animal findings support a role of supplementation with pantothenic acid (vitamin B5) in alleviating muscular fatigue whereas human data are still controversial. During exercise, rats made deficient in pantothenic acid became exhausted more rapidly and their tissue acetyl-CoA levels were lower than in vitamin B5-replete animals. A 2 week supplementation with 2 g/day of pantothenic acid translated into better performance for trained distance runners, with a better use of oxygen and less lactic acid accumulation, but this was not reproduced in another similar study (quoted in [121]). 

Folate (vitamin B9) supplementation has been successfully tested in patients with beta-thalassemia, an inherited disorder in which red blood cells and hemoglobin levels are affected, leading to fatigue and muscular weakness. In spite of some methodological drawbacks, it has been found that, in 73 thalassemic children aged approximately 10 years, folic acid supplementation (1 mg/day for 3 months) led to a reduction of reported fatigue perception [122]. 

#### 6.2.2. Supplementation with Vitamin C and Physical Fatigue 

Intervention trials assessing the effect of vitamin C on perceived fatigue have been limited and have not always used physiological doses or administration routes, and yet they confirm a favorable outcome. For example, 20 obese adults received per oral route either 500 mg of vitamin C or a placebo daily for 4 weeks. Ratings of perceived exertion during moderate exercise and general fatigue scores were significantly decreased in subjects receiving vitamin C [123]. In another study, 44 workers who received an oral dose of 6 g vitamin C daily for 2 weeks in an open trial reported lower perceived fatigue and exhibited significantly increased blood vitamin C, from 42.9 to 68.6 µmol/L [124]. These findings were confirmed when vitamin C was provided by the intravenous route, a non-physiological but relevant route of administration for research or medical purposes. In a randomised trial, 141 office workers aged 20–49 years received 10 g vitamin C or a placebo in one intravenous injection. Fatigue scores 2 h and 1 day after intervention were significantly lower in the vitamin C-treated group and especially in those who had the lowest baseline serum vitamin C. [125]., Vitamin C supplementation is currently used in patients who have cancer- and chemotherapy-related symptoms, such as fatigue, insomnia, loss of appetite, nausea, and pain. Several recent studies have indicated that intravenous vitamin C led to significant improvements of these symptoms and found that not only overall health, but also physical, role, cognitive, emotional, and social functioning were improved [126]. 

Vitamin C may also have an indirect impact on oxygen delivery to tissues. It can enhance iron absorption from vegetal sources and increase the mobilization of iron from body stores while providing protection against oxidative damage that may occur to red blood cells. When the level of antioxidants, including vitamin C, is low, this can result in hemolysis and blood loss through capillary hemorrhage, contributing to anemia [127]. 

#### 6.2.3. Supplementation with Iron and Magnesium and Physical Fatigue 

The effect of iron on fatigue has been addressed in a meta-analysis that identified six relevant randomised controlled trials evaluating iron supplementation in young premenopausal adult women with nonanemic iron deficiency. This study revealed a highly significant treatment effect of iron on fatigue, with an overall decrease of fatigue complaints of more than 60% [128]. Among the included studies, a 12 week trial of iron therapy (80 mg elemental iron daily) enrolled 198 women aged 18–53 years with unexplained fatigue, according to answers to a validated questionnaire in the practices of 44 French general practitioners. These women were menstruating, nonanemic but with low or borderline ferritin levels, denoting a marginal blood iron status. The mean group score for fatigue decreased in the placebo group (by 29%), but it decreased significantly more (by 48%) after iron intake in the treated group. Iron supplementation increased hemoglobin by 0.32 g/dL and ferritin by 11.4 µg/L, when compared with placebo [129]. Other trials, not included in this meta-analysis, reported similar findings: in a study that enrolled 290 women, fatigue was reduced significantly more (65.3%) in those who received a single infusion of 1 g of iron than in placebo-treated controls (52.7%) [130]. Iron supplementation thus helps in recovery from perceived fatigue above the placebo effect.

In theory, iron deficiency without anemia should not affect aerobic capacity because of the strong dependency of VO_2_max on oxygen transport, which is not impaired in nonanemic subjects [128]. However, a meta-analysis gathering data from 15 studies carried out in endurance athletes found a moderate but significant favorable effect on VO_2_max of iron intervention (oral iron in most studies, with dosages varying from 10 to 300 mg/day and for durations ranging from 14 to 84 days). The influence of iron treatment in nonanemic athletes should not be dismissed, as it may serve to prevent an otherwise detrimental reduction, which would place the athlete at a greater risk of developing iron deficiency anemia [131]. Furthermore, when endurance capacity (i.e., the maximum length of time an individual can sustain a given workload) and energetic efficiency (i.e., the amount of physiological energy required to perform a given amount of external work) are considered, the effect of iron supplementation can also be seen in milder iron deficiencies. A meta-analysis on 22 studies found that iron supplementation improved both maximal exercise performance and submaximal exercise performance, demonstrated by a lower heart rate required to achieve defined workloads [132]. In a recent trial, 73 young untrained women with nonanemic iron deficiency received either a placebo or 42 mg iron daily for 8 weeks and were submitted to aerobic exercise training; iron supplementation increased endurance performance at submaximal and maximal exercise intensities; serum ferritin increased significantly in the supplemented group vs. the placebo group (46.9 µg/L vs. 21.8 µg/L) [133].

This effect of iron deficiency on fatigue and work capacity has strong socio-economic consequences. Iron deficiency has been shown to be associated with lower work capacity for agricultural workers in several developing countries, including China, Colombia, Guatemala, Indonesia, Kenya, and Sri Lanka, in men and in women. Iron supplementation increased work capacity and output, with productivity gains, and take-home pay ranged from 10% to 30% above previous levels [127].

Magnesium can have an effect both on perceived physical fatigue and on muscle fatigue, that can affect physical performance. There is only a limited number of studies addressing the relationship between magnesium supplementation and physical fatigue. Supplementation with 400 or 800 mg magnesium daily for 4 weeks has been shown to reduce the perception of fatigue in 25 breast cancer women experiencing hot flushes in an uncontrolled pilot trial [134]. One intervention study in physically active college students found that endurance performance during submaximal exercise increased after magnesium supplementation (8 mg/kg/day, which is 500 mg/day approximately for a body weight of 60 kg), thus suggesting a possible beneficial impact on the perception of exercise-related fatigue [135]. This impact of magnesium on exercise-related fatigue may be related to a reduction/delay in lactate accumulation in muscles [115]. 

Randomised controlled trials that evaluated the impact of magnesium supplementation on muscle strength and exercise performance in adults have provided conflicting results, with some studies showing a beneficial impact [136,137], while others failed to report benefits [138]. For example, magnesium supplementation (8 mg/kg/day) for 7 weeks was shown to improve muscle strength (quadriceps torque) significantly in 26 untrained young adults submitted to a strength training programme, as compared with placebo [136]. In another trial, 1 week supplementation with 300 mg magnesium/day was reported to significantly increase muscle strength (+ 8% in bench press scores) as compared with baseline measurements, while no significant changes were observed with placebo, in 13 recreational sportspeople [137]. Data from animal studies suggest that magnesium might improve exercise performance via enhancing glucose availability in the brain, muscle and blood, and reducing/delaying lactate accumulation in muscles, which may delay exhaustion [115]. The positive impact on lactate has been observed in a controlled clinical study where magnesium supplementation (10 mg/kg body weight/day) for 4 weeks attenuated the exercise-induced increase in plasma lactate levels of 30 young Tae-Kwan-do sportsmen [139]. 

## 7. Clinical Evidence of the Role of Vitamins and Minerals on Mental Fatigue and Cognitive or Psychological Functions

### 7.1. Impact of Vitamins and Minerals Frank Deficiencies on Mental Fatigue and Cognitive or Psychological Functions 

#### 7.1.1. Deficiencies in Individual B Vitamins and Mental Fatigue and Cognitive or Psychological Functions 

Cerebral beriberi (thiamine (vitamin B1) deficiency) may lead to encephalopathies, characterized by abnormal eye movements, and cognitive impairment, involving a confused, apathetic state and a profound memory disorder, with severe amnesia and loss of recent and working memory. These symptoms are most often seen in people with heavy alcohol intake, but they have also been found in subjects with very low dietary intakes of vitamin B1 [99,140]. When thiamine deficiency is corrected early enough, symptoms can be fully reversed and significant brain damage can be prevented. However permanent brain damage and irreversible cognitive dysfunction will occur if thiamine deficiency persists and this can be critical as encephalopathies linked to thiamine deficiency remain largely undiagnosed [141]. Specific clinical neurologic symptoms are observed only when thiamine deficiency is severe, and it has long been believed that low thiamine status had no long-term sequelae. An accidental thiamine deficiency in children fed an infant formula erroneously lacking thiamine, resulted in persistent cognitive and motor deficits. Even the children who initially appeared unaffected subsequently displayed a range of cognitive dysfunction, including delayed language acquisition, language impairment and dyslexia, poor coordination skills, and learning disabilities; quoted in [99]. While this is likely to be related to neuronal injuries during the critical period of brain development, it can be hypothesized that subtle negative cognitive outcomes resulting from lifelong subclinical thiamine deficiency may also occur in populations with low dietary thiamine intake. Studies in animal models have shown that thiamine deficiency leads to memory impairment: performance in several cognitive tests, including avoidance or maze tasks, is decreased in thiamine-deficient rats compared with control animals. In addition, diminished learning was seen in animals with thiamine deficiency, which exhibited reduced neurogenesis, a feature also encountered in the early stages of Alzheimer disease [142].

Long-term niacin (vitamin B3) deficiency can lead to neurological symptoms, including depression and loss of memory [102]. These symptoms attenuate or disappear following niacin intake, confirming its role in the brain and nervous system [143]. In addition, niacin receptors, which are normally distributed throughout the brain, are affected in several brain areas in people with Parkinson disease (who generally have low niacin levels); quoted in [94]. 

Deficiency in pantothenic acid (vitamin B5) results in neurological dysfunction. Dogs treated with an antagonist of vitamin B5 that induces a deficiency of pantothenic acid developed an acute encephalopathy [144]. In humans, there is a rare genetic defect in the production of pantothenate kinase, an enzyme that is crucial in the synthesis of acetyl-CoA, which translates into inherited neurodegenerative diseases or neurodegeneration [145]. Experimentally induced deficiency in pantothenic acid results in personality changes [103].

During severe pyridoxine (vitamin B6) deficiency, disturbed neurologic functions, such as convulsive epileptic seizures and irritability and depression, are observed [146]. 

Biotin (vitamin B8) deficiency is associated with neurological disorders: depression, lethargy, hallucinations, and paraesthesia (tingling/burning sensation) of the extremities [49]. The mechanisms leading to these neurological disorders are not well known, but it could reasonably be assumed that the key role of biotin in the energetic metabolism of all cells, including in brain and nerves, participates in the normal functioning of the nervous system [29].

Cobalamin (vitamin B12) deficiency induces neurological dysfunction, including myelopathy, neuropathy and neuropsychiatric abnormalities. Such neurological damage has been reported in 20–30% of cases of vitamin B12 deficiency [147], and can even occur before serum vitamin B12 concentrations reach a level that would be considered deficient by standard criteria. It is likely due to progressive neuronal degeneration, due to the inhibition of methionine synthase [51]. In addition, deficiencies in vitamin B12 are associated with mental and cognitive impairment, such as irritability, memory loss, depression, and cognitive disturbances up to dementia [148].

#### 7.1.2. Deficiency in Vitamin C and Minerals and Mental Fatigue and Cognitive or Psychological Functions 

Scurvy (vitamin C deficiency) is frequently associated with depression, hypochondria, and mood changes, and may be related to deficient dopamine hydroxylation. Because the brain consumes a lot of oxygen and is very rich in polyunsaturated fatty acids, which are prone to oxidation, there is a strong need for antioxidant molecules, such as vitamin C. Oxidative stress has a role, especially during ageing, in the development of cognitive problems in working memory, reasoning, or problem solving [149]. Early-life iron deficiency may produce irreversible neural changes when neurogenesis and differentiation of brain regions are occurring; numerous observational studies have shown associations between iron deficiency and/or associated anemia and poor motor and cognitive development outcomes in children. Findings from prospective studies indicate that infants who are anemic have poorer cognitive capacities, impaired school achievement, and increased behavioural difficulties into middle childhood [150].

### 7.2. Relationships between Vitamin and Mineral Dietary Intake or Status and Mental Fatigue and Cognitive or Psychological Functions

Beyond severe deficiencies, which readily translate into clinical consequences, inadequate status or intakes in vitamins and minerals is known to be associated with cognitive and psychological dysfunction. While the general adult population is affected, studies are especially numerous in populations where the brain is either more demanding because of its rapid development, such as in children, or is affected by degeneration, such as in older subjects. This section and the following one (observational studies, then supplementation studies) report data on all these age ranges.

#### 7.2.1. Intake or Status in Individual B Vitamin and Mental Fatigue and Cognitive or Psychological Functions

Low dietary intakes of thiamine (vitamin B1) are associated with higher risk of cognitive impairment; thiamine-dependent biochemical processes are diminished in patients with Alzheimer disease and glucose metabolism is especially hampered [142]. 

In 100 elderly Koreans with mild cognitive impairment, a higher riboflavin (vitamin B2) intake was correlated with better cognitive performance on several tests [151]. 

Positive associations have been found between niacin (vitamin B3) status and the risk of Alzheimer disease and cognitive decline [152]. In addition, in a cohort of 3136 American adults followed for 20 years from age 18 to 30 years, those in the highest quintile of dietary niacin intake at baseline (i.e., a median intake of 24.7 mg/day) had better results in cognitive tests 20–25 years later than did those with the lowest dietary intake of niacin (median 8.6 mg/day, so below the US recommendation, set at 15 mg daily) [153]. In 317 healthy Korean children with no previous diagnosis of neurologic or psychiatric disorders, analyses showed that fewer omission errors were made during tests in those with the highest vitamin B3 intakes [154]. 

Positive correlations exist between low dietary intakes of pantothenic acid (vitamin B5) (together with other macronutrients and micronutrients) and low scores obtained by 97 adults with mood disorders in the Global Assessment of Functioning, a questionnaire designed to evaluate social, occupational, and psychological functioning [155]. Similar findings come from a study in 710 Spanish school children that suggest that children with depressive symptoms had lower intakes of vitamin B5, as well as of other B vitamins [156].

A negative relationship has been found between depressive symptoms and the levels of intake of B vitamins, including pyridoxine (vitamin B6) in 914 elderly Japanese females, but not in 720 males [157]. In addition, a cohort of 3136 young adults has been followed for at least 20 years from age 18 to 30 years and those in the highest quintile of dietary pyridoxine intake at baseline (i.e., a median intake of 3.0 mg/day) had better results in psychomotor speed tests 20 to 25 years later than those with the lowest dietary intake of pyridoxine (median 0.7 mg/day) [153].

Few data have been found on the association between biotin (vitamin B8) intake and potential neurological disorders. A cross-sectional observational study in Spain reported a significantly lower dietary intake of biotin in 710 children (6–9 years old) with depressive symptoms compared with those without depressive symptoms [156]. However, intakes of other nutrients, such as those of zinc and vitamin B12, were also significantly lower. 

Low folate (vitamin B9) blood status and/or low dietary intakes of folates are associated with psychological symptoms such as depression and anxiety in adults and elderly subjects. In 2266 Japanese adults (mean age 43.5 years), those with depressive symptoms were found to have lower dietary energy-adjusted folate intakes than those without depressive mood [158]. In 425 Iranian young adults (mean age 32.5 years), those with folate intake above the recommendation had significantly lower scores when assessing depressive affect, somatic complaints and interpersonal difficulties [159]. In a cohort of more than 2200 healthy Norwegian individuals aged 71–75 years, high plasma folate levels were associated with improved cognitive performance [160]. Such associations have been confirmed in two recent meta-analyses. The first one found a small but significant association, such that individuals with depression had lower folate levels than those without depression [161]. In the second one, gathering 6949 elderly subjects, a higher risk of depression was associated with low folate status [162]. Additional evidence comes from longitudinal studies, such as in a prospective cohort that enrolled more than 7000 women aged 65–79 years: low baseline folate intakes (i.e., below the recommended 400 µg/day) or low serum folate concentrations were significantly associated with a higher risk of cognitive decline or dementia during a 5 year follow-up [163]. Similarly, young adults with a high dietary intake of folate (mean intake: 384 µg/day) had better cognitive function 20 to 25 years later, in midlife, when compared with those with the lowest folate dietary intakes (mean intake: 152 µg/day) [153]. 

The elderly and young children have been especially studied regarding their neuropsychiatric and cognitive functions in relation to cobalamin (vitamin B12) status [164,165,166], while studies in adults are scarce. The importance of early supply of vitamin B12, during pregnancy and infancy, on later cognitive outcomes has been recently reviewed [164]. Higher maternal vitamin B12 status and/or higher vitamin B12 intakes during pregnancy have been prospectively associated with improved measures of cognition in 330 children later in life. In Nepalese children aged 2–12 months, the vitamin B12 status was biochemically assessed and was found significantly associated, 5 years later, with improved development and performance on assessments of social perception and visuospatial abilities [167]. In 72 adolescents aged 10–16 years, it has been shown that the cognitive performance was worse when they had consumed a vegan diet (thus containing low amounts of vitamin B12) from birth to 6 years of age, when compared with omnivorous children [168]. Cross-sectional studies in older children or adolescents most often found that lower biochemical vitamin B12 status was associated with impaired school performance and mental and social development, as well as short-term memory and attention [164]. These findings are suggestive of a role for vitamin B12 in enabling improvement of cognitive capabilities in children and are consistent with its role in neural myelination, brain development, and fetal and child growth. In addition, a higher intake of B vitamins, including cobalamin, throughout young adulthood was associated with better cognitive function in midlife. A cohort of 3136 young adults has been followed for 20 years from age 18 to 30 years and data show that those in the highest quintile of dietary vitamin B12 intake at baseline (i.e., a median intake of 8.7 µg/day) had better psychomotor speed 20 to 25 years later than those with the lowest dietary intake of cobalamin (median 2.2 µg/day) [153]. Several studies using functional biomarkers of vitamin B12 status showed a trend towards associations between poor vitamin B12 status in an ageing population and faster cognitive decline and risk of Alzheimer’s disease [169]. A population-based study in 3884 elderly men and women with depressive disorders found that those with vitamin B12 deficiency were almost 70% more likely to experience depression than those with normal vitamin B12 status [170]. However, more recent systematic reviews have less clear conclusions: no association was found between blood vitamin B12 concentrations and cognitive decline or dementia, but four studies that assessed vitamin B12 status with newer biomarkers found associations between low vitamin B12 status and an enhanced risk of diagnosis with dementia or cognitive decline [165]. 

The role of vitamins B6, B9 and B12 on cognition may be linked to their close biochemical interplay (Figure 2). A deficiency in any of these vitamins may lead to a lack of methionine synthesis that leads to the accumulation of homocysteine, which is a suspected risk factor for cognitive dysfunction and dementia, although this point remains controversial [94,171,172].

#### 7.2.2. Intake or Status in Vitamin C, Iron, Magnesium and Zinc and Mental Fatigue and Cognitive or Psychological Function

Several observational studies have suggested that a low plasma level of vitamin C or low vitamin C intakes were correlated to an increased incidence of depression or anxiety syndromes in various populations. In India, vitamin C levels were lower in subjects with generalized anxiety disorder versus subjects without the disorder: in a study in 80 subjects aged 20–60 attending a psychiatric clinic, dietary supplementation for 6 weeks with 1 g vitamin C daily led to a significant reduction in anxiety and depression scores [173]. Vitamin C status has been found to be associated with better mood in male students from New Zealand: the higher the plasma concentration, the lower the reported mood disturbance, depression, confusion and anger, assessed by the Profile of Mood scale and subscales [174]. In a case-control study in people aged over 60 years, vitamin C intake from foods was above the recommended amount in all subjects, but was significantly lower (109 mg/day) in depressive subjects versus non depressive ones (143 mg/day) [175]. 

There is a wide consensus regarding the critical importance of appropriate iron supply during neurodevelopment from birth, and even before, up to the first years of life: the neurocognitive damage elicited by iron deficiency during this period is irreversible and will persist into adulthood [176]. However, inadequate iron intakes or status later in life may also translate into cognitive impairment: when compared with controls with a normal iron status, 28 young Egyptian adults who displayed low iron status (mean blood hemoglobin approximately 7 g/dL) had significantly lower cognitive performance, assessed via the Mini-Mental State Examination, but also via the Wechsler memory and intelligence scales [177]. A study in 149 American young women aged 18–35 years found that iron-sufficient women performed better and more rapidly on cognitive tasks than did the women with iron deficiency anemia (hemoglobin levels between 10.5 and 11.9 g/dL and two abnormal iron status values). Attention, memory and learning were affected and data show that cognitive performance decreased when the severity of iron deficiency increased [178]. In another study, 428 Chinese adolescents 12 years of age and with a low iron status (below 75 µg/dL) had slower performance in tasks that measured abstraction and mental flexibility and lower spatial processing abilities compared with those with normal iron status. However, adolescents with excessive blood iron (above 175 µg/dL) displayed a lower accuracy in the spatial processing ability task and a longer reaction time when abstraction and mental flexibility were assessed, suggesting the existence of a safe window for iron benefits [171]. Overall, there is evidence that the negative cognitive consequences of iron deficiency are not limited to infancy and childhood. 

Conversely, there are many indications that iron overload and presence of unbound iron in cellular structure have deleterious consequences on mental health. Indeed, excessive iron deposits become prevalent in the cerebral region upon ageing and even more so in the brains of subjects suffering from Parkinson disease [179] or Alzheimer disease [180] diseases, where they are associated with the neurodegeneration observed in these pathologies. 

Magnesium status and intakes have been associated with the risk of dementia: in a prospective study in 9569 Dutch people healthy at baseline, a low serum magnesium level (below 0.79 mmol/L) was associated with a higher risk of dementia 8 years later (hazard ratio 1.32; 95% confidence interval 1.02–1.69). Noteworthy, a similar risk increase was also associated with the highest magnesium serum concentration (above 0.90 mmol/L), suggesting the existence of an optimal status in between [181]. According to meta-analyses, both insufficient dietary magnesium intakes [182,183] and low magnesium biological status [184] are associated with a higher risk of depression. For example, a recent study in 3600 subjects with a mean age of 62 years observed that those with a high serum magnesium were less likely to be depressed [185]. In a review of observational studies comparing magnesium status in a total of 559 subjects suffering from Alzheimer disease, a lower magnesium concentration in the cerebrospinal fluid was found, when compared with 381 healthy matched controls [186]. Hypomagnesaemia has also been associated with other types of neural dysfunction, possibly including those observed in alcoholism [187]. Indeed, magnesium is known to reduce the intensity of addiction to alcohol and other substances and alcohol abusers have lower plasma and intracellular magnesium concentrations, compared with healthy subjects [188]. 

Observational studies show an association between low **zinc** dietary intake or low zinc status and depression in adults or elderly subjects [189,190,191]. In a meta-analysis of nine studies, a high dietary zinc intake appears to be linked to better protection against depression, with a meaningful relative risk of 0.67 (95% confidence interval: 0.58–0.76); associations were significant both in cross-sectional and in prospective studies [192]. Zinc deficiency is suspected to be a mediator of toxic effects of alcohol abuse, including at the brain level, and zinc status is generally low in alcoholic patients [193]. As for iron, zinc overload may be deleterious to brain functions: in patients suffering from Alzheimer disease, zinc at physiological levels suppresses β-amyloid-induced neurotoxicity by selectively precipitating aggregation intermediates; however, at high levels, the binding of zinc to β-amyloid may rather enhance fibrillar β-amyloid aggregation, leading to neurodegeneration [194].

### 7.3. Effect of Supplementation with Vitamins and Minerals on Mental Fatigue and Cognitive or Psychological Functions

#### 7.3.1. Supplementation with Individual B Vitamins on Mental Fatigue and Cognitive or Psychological Functions

Thiamine (vitamin B1) supplementation could be effective in preventing cognitive impairment in at-risk situations, i.e., before cognitive dysfunction occurs. In a retrospective cohort study in more than 10, 000 Taiwanese patients with a recently diagnosed alcohol use disorder, those who received thiamine therapy after the diagnosis were compared with a control group without thiamine; the treated group had a lower hazard ratio (0.76; 95% confidence interval: 0.60–0.96) of dementia [195]. Recently, the administration of a synthetic derivative of thiamine (300 mg/day for 18 months, orally) in five patients with mild to moderate dementia resulted in improved cognitive ability measured via the Mini-Mental State Examination [196]. An earlier study had assessed the effects of a high dose (50 mg daily for 2 months) of thiamine or placebo on 120 young adult women with adequate thiamine status at the study outset. Thiamine status, assessed via transketolase activation, was improved in treated subjects and this was associated with improved attention and a trend toward improved mood [197]. 

Supplementation with 250 mg niacin (vitamin B3) was found to attenuate the disturbed sleep architecture associated with Parkinson disease, probably through a normalization of the function of niacin receptors; quoted in [94]. 

Findings regarding the effect of folate (vitamin B9) supplementation on cognitive outcomes are mixed but globally indicative of a favorable effect. In a long-term supplementation trial 818 healthy adults, aged 50 to 70 years, received daily for 3 years either folic acid (800 µg/day) or placebo; their folate dietary intake was lower than recommended (median intake approximately 185 µg/day) but did not differ between groups. When compared with the placebo group, the treated group had significant improvements in serum folate, which increased from 12 nmol/L at baseline up to 53 nmol/L after 1 year and 76 nmol/L after 3 years of supplementation, and this increase was associated with improvements in several cognitive domains such as global cognitive function, information-processing speed and memory storage [198]. In another smaller trial, 30 elderly patients were given 15 g folic acid or a placebo for 60 days; treated patients experienced improved attention efficiency and memory processes [199]. Conversely, no effect of 5 mg/day of folic acid was found in another trial in 24 healthy elderly subjects. However, these subjects had a normal cognitive function and normal blood folate levels at baseline, suggesting that they may not have been in a position to benefit from supplementation [200]. Oral folic acid (400 µg/day), associated with vitamin B12 (100 µg/day) was provided during 2 years as a supplement to 752 community-dwelling older adults, aged 60 to 74 years, with depressive symptoms. The vitamin-treated group displayed significantly better cognitive functioning, especially in immediate and delayed memory performance, when compared with the control group [201]. Similarly, in a recent randomised controlled trial in 180 subjects with mild cognitive impairment, those having received folic acid (400 µg/day) for 2 years exhibited better cognitive performance (full scale and verbal intelligence, memory) when compared with the placebo-treated group [202]. As a whole, studies suggest that supplementation with folic acid can be effective for improving cognitive function and this can be seen especially in population with low baseline folic acid status, with some degree of cognitive impairment and who are supplemented for a duration sufficient to restore their folate status. 

#### 7.3.2. Supplementation with Vitamin C, Iron, Magnesium or Zinc and Mental Fatigue and Cognitive or Psychological Functions

Intervention trials have shown that vitamin C could decrease symptoms of anxiety [203], and a recent report of three cases indicated that, in alcoholic men with latent scurvy associated with extreme lassitude, supplementation with high doses of vitamin C (500 to 1000 mg/day for 3 months) allowed the regression of the symptoms, greatly improved the quality of life, and gave the possibility of a return to work [204].

In spite of the role of iron in neurodevelopment, iron supplementation studies addressing motor and mental development outcomes in young children (below 5 years of age) have shown inconsistent effects on development [127]. Effects are clearer in older children, as shown in a meta-analysis of 32 studies that included 7089 children aged 5–12 years. Iron supplementation in anemic children significantly improved measures of attention and concentration, global cognitive scores and intelligence quotient [205]. Increasing the iron intake in adolescents has also been shown effective in improving their cognitive performance. For example, attention and memory performance increased significantly in 140 Indian adolescents aged 12–16 years who consumed for 6 months a biofortified iron-rich millet compared with conventional millet, which increased their ferritin level vs. placebo (30 vs. 22 µg/L) [206]. Another study, in 428 Chinese adolescents aged 12 years, found that either iron deficiency or excessive iron levels contribute to reduced neurocognitive performance in a domain-specific manner [171], indicating that a balance should be found. Indeed, several lines of evidence suggest that excessive iron accumulation can be detrimental to brain structures and could be associated with brain ageing and neurodegenerative disorders [207]. Several studies in adults have suggested that reduced iron status has a detrimental impact on cognitive functions. A systematic review has identified ten supplementation trials in premenopausal women, a population at higher risk of iron deficiency because of enhanced requirements. Seven of these studies found that iron supplementation improved several dimensions of mood and cognition, including memory and intellectual ability [208]. Following iron supplementation (60 mg daily for 4 months), 149 young American women with iron deficiency anemia at baseline experienced a significant improvement in serum ferritin (22.8 µg/L vs. 9.2 µg/L in the placebo group) that was associated with a 5-fold improvement in cognitive performance [178].

Anxiety and depression are mediated by dysfunctional glutaminergic neurotransmission and there is thus a rationale for an effect of magnesium on these outcomes. Supplementation studies with magnesium alone or in combination with other nutrients has been recently reviewed and the conclusion has been, in spite of some methodological weaknesses, that magnesium can be a useful adjunct to manage mild anxiety symptoms [209]. For example, magnesium lactate (300 mg), associated with vitamin B6, was found as effective as a pharmacological anxiolytic in improving anxiety scale scores in mildly anxious subjects [210] and a recent intervention trial found that in 264 healthy stressed subjects with low magnesaemia, supplementation with 300 mg magnesium daily resulted in reduced stress scores in a few weeks. In the most severely stressed subjects, the combination of magnesium with vitamin B6 was more effective than magnesium alone, possibly through vitamin B6 facilitating cellular uptake of magnesium [211]. Similarly, depressive symptoms can also be mitigated by appropriate magnesium supplementation, as suggested by a recent meta-analysis in which more than half of the studies indicated a decreased risk of depression with higher magnesium intakes, the largest risk reduction being observed with 320 mg/day [183]. This has been confirmed in a recent randomised controlled trial in which 60 depressive and hypomagnesaemic subjects received either 250 mg of magnesium or a placebo for 8 weeks. Depression scores improved significantly in those who received magnesium when compared with the placebo, while serum magnesium was slightly increased in the treated group (+ 0.31 mg/dL) vs. the control group (+ 0.09 mg/dL) [212]. 

Clinical studies assessing the effect of zinc on humans’ cognitive outcomes are less numerous and do not provide a clear-cut picture. Meta-analysis of data from intervention trials in children found no significant overall effect of zinc intake on any indices of cognitive function. However, this meta-analysis had included heterogeneous trials with some in which mothers were supplemented during pregnancy and others in which zinc supplementation was provided to the children. When focusing only on the latest trials, significant improvements were seen in children’s executive and motor functions [213]. Among the few studies addressing the relationship between zinc and cognition in adults, one had tested zinc supplementation (0, 15, or 30 mg/day) in 387 healthy adults aged 55–87 years, and found a beneficial effect of either zinc dose on spatial working memory, but not on several other measures [214].

#### 7.3.3. Effect of Supplementation with Multiple Micronutrients on Mental Fatigue and Cognitive or Psychological Functions

There is thus ample clinical evidence for a role of selected individual vitamins and minerals in contributing to enhance mood, well-being and cognitive functions as well as to reduce symptoms of mental fatigue. In relation to the numerous interplays between vitamins and minerals, and with the observations that inadequate intakes or status are often co-existing for several micronutrients within the same subjects, numerous supplementation studies have been performed with combinations of vitamins and minerals, which varied by the number, associations and dosages of micronutrients, as well as by the duration and addressed populations and outcomes. It is beyond the scope of this review to attempt assessment of their results, however, a few systematic reviews or meta-analyses in the area can be highlighted. Published in the last few years, they most often confirmed favorable effects for micronutrient interventions. 

Cognitive performance, and especially fluid intelligence, appeared improved following multiple micronutrient supplementation in an analysis of 18 randomised controlled trials in school-aged children [215], confirming an earlier meta-analysis in this age group [216]. In adults, the available data suggest an improvement of psychological and cognitive functions following multiple supplementations, based on several reviews [94,217] and on meta-analyses of intervention trials addressing mood and psychological state [218,219]. Regarding mood, the most recent meta-analysis found that in 11 out of 18 trials a combination of B vitamins resulted in improvement of mood when compared with a placebo. Benefits on depressive symptoms failed to reach statistical significance, but conclusions were stronger for stress. Supplementation was more efficient in populations with a poor mood status or a poor nutrient status at baseline [219]. To illustrate this conclusion, one trial has shown that, compared with placebo, a 4 week supplementation (B vitamins, vitamin C, zinc, calcium and magnesium) in 58 healthy adults was associated with a significant improvement of mood, in relation to increased levels of B vitamins and lowered homocysteine in blood [220]. Interestingly, the same authors also reported preliminary findings suggesting that this same 4-week multiple micronutrient supplementation could induce changes in brain function, using both hemodynamic and electrophysiological assessments. During working memory tasks, the centro-parietal regions showed higher activity after multiple supplementation, especially when subjects were investigated in a situation of mental fatigue [221]. The enhanced benefit of supplementation for subjects with low vitamin status at baseline is supported, for example, by the results of a 13 years supplementation trial of B vitamins (B6 + B9 + B12) in which cognitive function was assessed in women with a high cardiovascular risk. No cognitive improvement was shown in the whole population of 5 442 women, but a sub-analysis in those women who had a low baseline dietary intake of B vitamins (less than 1.9 mg/day for vitamin B6 and less than 279 µg/day for vitamin B9) showed that supplementation preserved cognitive capacities in this group at the 13 year assessment [222]. 

Supplementation studies with multiple vitamins and minerals are sometimes difficult to interpret, but they are clinically very relevant. Isolating the effect of a single component is often clinically difficult: nutrient deficiencies, especially mild ones, often result in nonspecific symptoms and they tend to result from unbalanced food intakes, which are likely to lead to inadequate intakes in multiple nutrients. 

## 8. Conclusions

There is a strong biological and physiological rationale that indicates that the long-known involvement of vitamins and minerals in cellular energy production translates into functional and physiological outcomes in humans, including perceived physical and mental fatigue as well as psychological and cognitive functions. Indeed, the organs supportive of these functions, skeletal muscle and brain, are the most energy-demanding ones in the human body. In addition, vitamins and minerals, especially the B vitamins, vitamin C, iron and magnesium addressed in this review, are mandatory to extract this energy from food and present it in a physiologically usable form. Furthermore, because there is a close interplay between these micronutrients across the successive steps of energy production, all of them should be available simultaneously as the whole system may be slowed down by a lack in a single one of them. While this role in energy production is pivotal, other functions fulfilled by micronutrients are also key in relation to fatigue and cognition. Besides energy, muscles and brain also need oxygen, which should be delivered by the hemoglobin present in red blood cells; this process requires an efficient erythropoiesis, for which iron and also several B vitamins are critical. Inadequate intakes in these micronutrients result in anemia, with resultant fatigue and weakness symptoms. Oxygen is mandatory, but also generates oxidative reactive species, which are potentially harmful so need to be controlled by the anti-oxidative defences of the organism, in which vitamin C plays a key role. Furthermore, many vitamins and minerals have a role in the synthesis of neurotransmitters, development and maintenance of neuronal membranes, and brain receptor modification, with obvious consequences for brain activation and thus cognitive function. 

Clinical confirmation of the essential nature of micronutrients and of their role in alleviating fatigue and cognitive dysfunction comes from observations in subjects with low or very low intakes or deficient biological status and from reversal of symptoms following supplementation. Severe deficiencies are no longer common, but there is some evidence that sub-clinical or marginal deficiencies can have negative consequences on physical and mental fatigue, as well as on cognitive and psychological functions. This is confirmed by supplementation studies, although many are enrolling subjects without a clear assessment of their baseline nutritional status, so the populations may include subjects both with and without a deficient or sub-deficient status. In such cases, it may happen that the supplementation is not effective, because a significant proportion of subjects already has a sufficient micronutrient intake and will not benefit from the supplementation. However, when restricted to subjects with inadequate intakes or status, conclusions are most often favorable. 

Supplementing individuals with vitamin and minerals is thus highly likely to result in health benefits in the areas of mental and physical fatigue, as well as cognitive and psychological functions. However, efficient and safe supplementation should carefully consider practical dimensions, such as the chemical form of vitamins and minerals, which can affect their bioavailability, or the duration of treatment, which should be sufficient to ensure the restoration of adequate status in people where intakes are not sufficient, and who are the more likely to benefit from supplementation. In addition, caution should be exerted to avoid side effects and deleterious consequences of overdosing. It is for example known that neurotoxicity can be observed following excessive doses of vitamin B6, approximately 1 g daily, whereas recommended intakes are in the mg range [223]. Public Health Authorities have reviewed the existing evidence about health hazards associated with excessive intakes of all vitamins and minerals and have provided guidelines about tolerable upper limits of daily intake of vitamins and minerals that should be followed [223]. In most cases, remaining within the order of magnitude of the recommended daily allowance will provide the needed safety level. 

In this review, detailed information has been provided for 12 micronutrients, which highlights that consuming adequate intakes of vitamins and minerals contributes to maintenance of normal cognitive function and to enhanced well-being by decreasing perceived mental and physical fatigue and favoring positive mood. Health professionals are often confronted by complaints about fatigue and perceived psychological or cognitive difficulties. Once pathological aetiologias have been discarded, it is relevant to question the supply and status of vitamins and minerals and to provide nutritional advice first aiming at promoting balanced food choices, then focusing on the use of vitamin and mineral supplementation.
